# Case of Inflexibility of the Articulations, Attended with Great Pain, Relieved

**Published:** 1808-01

**Authors:** Rowland Bayley

**Affiliations:** Surgeon. Newton-Baschurch, Shropshire


### CASE *of* INFLEXIBILITY *of the* ARTICULATIONS, *attended with great Pain, relieved.*

JOHN LEEK, the subject of the following case, was a boy of perfect health, and particularly active till he attained the age of eight years, when (in Sept. 1795), he was attacked by a fever, accompanied with much inflammatory diathesis, with a truly *petechial* eruption upon the face, and all over the body; and with such excruciating pain in the limbs, especially in the joints, as to require the same to be rubbed for three or four hours at a time, and that frequently in the day and night; these frictions giving him some relief. The wrists and joints of the fingers were enlarged, but all the other articulations were free from swelling; nor was there, in any part, the least discoloration, independent of the *petechiæ*; there was also a destruction of all voluntary action, from head to foot. At that time I attended him, but my exertions (which, however, were not well seconded) proved of little avail. The complaint, in some respects, very much resembled acute rheumatism, but it differed from that disease in other particulars; in about a fortnight the fever subsided, and the eruption disappeared; the warm bath was then used, but the pain and loss of motion still continued, and with aggravating circumstances; the pain proceeding to the anterior part of the thorax, where it was oftentimes very distressing: he was carried from place to place, being himself unable to move; his nights were extremely tremely miserable; he enjoyed neither sound sleep, nor rest; and in this comfortless state his parents largely participated, as one or other of them was obliged to go to him to change his position, and to rub the parts mostly in pain, very often in the course of every night, and not unfrequently, even so many as ten times: he derived some support from two pillows, one being placed under the hams, and the other between the knees, nor could he lie without them. In this way he existed, somtimes enjoying about a month's suspension of pain, aud at others being tormented by it for as great a length of time, and such were the anxious and laborious services of his friends for many years: the febrile symptoms and petechial eruption also occasionally appeared, at which times his pains were always worse: this was particularly the case in the winter seasons. When he had been afflicted five years, he was able to support himself upon crutches. Notwithstanding every attempt to recover him, that was in the power of his parents (quibus res angustæ domi") had been made, (but I believe those attempts were chiefly or entirely empirical) he continued in the same deplorable state till February 1806, when my friend, Dr. Darwin, of Shrewsbury, and myself, had a good deal of conversation about him. The young man was then between eighteen and nineteen years old: there was at that time some little deformity about the knees, whose action was exceedingly confined; the legs and thighs were very diminutive; his elbows had so little extensiveness of motion, that he could not bring his left hand nearer to his mouth than twelve inches, nor the right than six; the joints of the fingers, on both hands, were nearly inflexible; the right index finger was permanently fixed over the adjoining one; and the spine was curved: but instead of minutely describing the several flexures of the different parts, I will just remark, that (making allowance for the want of approximation to the head by the arms) the posture of the body would not have been very unaptly referred to that which the fœtus generally observes in the uterus,* and to which

* Adductis ad abdomen genibus, flexis retrorsum cruribus, pedibus decussatis, manibusque sursum ad caput sublatis, quarum alteram circa tempora vel auriculas, alteram ad genam detinet; spina in orbem flectitur; caput ad genua, incurvato collo, propendet. Tali membrorum situ, qualem in somno per quietem quærimus.

Harvey, Exercit. de Partu.

which extreme old age again so naturally tends; so that he was only four feet three inches in height. But his sufferings were still the same, and still required the same constant attention of his friends, day and night: at this time he had the power of moving upon his crutches, and upon the points of his toes, but not more than a few yards at a time. Hopeless as the case appeared, Dr. Darwin encouraged me in my design of giving a fair trial to the *muriate of lime,* and *bright iron filings:* I therefore determined upon the following plan.

Of a solution of eight ounces of dry *muriate of lime,* in a pint of water, I directed him to take one tea spoonful in a tea cup full of small beer, twice a day, (at eleven and seven o'clock) for four weeks; increasing the dose to so much as his stomach would bear. When the four weeks should be completed, he was to take ten grains of *bright iron filings,* twice a day also, (at the same hours) for a fortnight, at the end of which time he was again to return the solution for a month longer; and this period to be again succeeded by another fortnight's course of the bright filings; and thus alternately to go on with these medicines for six months.

The patient began upon the solution on the nineteenth of February, 1806, and soon increased the quantity of it to *two* tea spoonfuls; and in a few weeks afterwards to *three;* but this at first excited considerable nausea; he, however, persevered, and became able to take it in this augmented dose, for the remainder of the term: but the quantity of iron-filings was not enlarged.

After he had taken the medicines a little more than a month, he could turn himself in bed; in another fortnight or three weeks he was enabled to lie without the pillows, and their use was left off; about the same time, he dispensed with the nightly frictions, and attendance of his friends, who, from that period have never been once disturbed; when he had pursued the course prescribed for two or three months, he found his pains very considerably relieved, and long before the completion of the six months, he entirely lost them. He was apprehensive that the ensuing winter would bring on a return of his sufferings, but he happily escaped, and has felt no relapse since his pains first gave way. The action of his arms is not improved, but his knees have a more extensive one; and that of the spine is greatly recovered: so that the curved outlines are become more rectilinear, and the body, consequently, much taller: but the best way for me to express the

*quantum*

*quantum* of these improvements, will be, to state the increase of height at different periods during the medicinal course; and this I am enabled to do from his own manuscript;* which, as, in addition to the above, it contains a short account of his relief, I will wholly transcribe.

"Midsummer 1805. Walked one inch higher only, than I had done for many years before. On 12th of February 1806, began to take the medicine, and in April had my crutches lengthened one inch and half; and in the course of fourteen days more, had them raised two inches more; and in the beginning of June, above two inches more: making in the whole, near six inches in rise. Before 1806, and in the beginning of that year, suffered much pain on the chest and shoulders, which would continue sometimes about a month, and at other times less than a month, and afterwards would fly into the thighs and knees, and be so violent that I could not walk at all for about a week; and after a while the pain would go off, and I should be as long free from pain, as I had been in pain; and then, or about that long, it would come on again, and be as before. I find myself better in every respect, having had none of those pains since I took the medicine: I find my joints more flexible, and being free from pain, am much stronger, and walk a great deal better." His crutches now, (Nov. 1807,) want raising another inch and half.

Although he is still obliged to be carried up and down stairs, he is able to travel upon his crutches several hundred yards without resting.

Thus, perfect relief from pain, undisturbed nights, and inexpressible comfort, have been restored to himself, and to his friends; and I am induced to publish the particulars for the benefit of others labouring under similar distress.

This young man possesses a very amiable disposition, and has great ingenuity in copying drawings, colouring maps, making chimney ornaments, &c. which he does in an exquisite manner; he writes a fine hand, holding the pen, or pencil, but in a most aukward position, between the thumb and distorted index finger of the right hand, the whole of the parts concerned in writing, &c. possessing only a very obscure motion.

I should

---

* This was written at my request, to inform me on some of the points contained therein, and without even the most distant idea that any further use was to be made of it. I had enjoined him to be as concise as possible, which accounts for the omission of those *minutiæ* which I have detailed.

I should have observed, in a more proper place, that the solution had the effect of giving a temporary stimulus to his appetite (which had always been pretty good), in a very considerable degree, every time he took it;—and that the use of the medicines was regularly persevered in, for the six months, except upon two or three occasions, when it was obliged to be suspended for a few days, or a week, on account of catarrh, which rendered him quite unable to take them; nor could they, at such times, be with propriety advised. The *muriate of lime* produced no other sensible effects, than those already mentioned; namely, *nausea* upon beginning on a large dose, and *increase of appetite*. He took the *filings* in treacle.

No notice has been taken of his pulse, nor of some other circumstances usually attended to; because nothing particular occurred, either in the vital or natural functions.

He was, when he first became the subject of this treatment, only four feet three inches high. I measured him myself a few days ago, and found his height to be four feet eleven inches and a half.

I am, &c.

ROWLAND BAYLEY, Surgeon.

*Newton-Baschurch, Shropshire,*

